# *Echo Research and Practice* enters a new era

**DOI:** 10.1186/s44156-022-00007-4

**Published:** 2022-07-13

**Authors:** Mark Monaghan

**Affiliations:** King’s College Hospital NHS Foundation Trust, Denmark Hill, London, UK

As *Echo Research and Practice (ERP)* beds down with its new publisher (BMC part of Springer Nature) I’m excited to bring you news of some important changes within the Editorial Board [1].

Sadly, after many years of amazing service to both *ERP* and the British Society of Echocardiography (BSE), Prof Nihoyannopoulos has decided to step down as Editor-in-Chief of the Journal. However, I’m pleased to report that he will remain an active member of the Editorial Board and I’d like to thank him for all his hard work and guidance that has helped make the Journal a success.

Having previously been Deputy Editor-in-Chief, I’ve been asked by the BSE to take on the role of Editor-in-Chief, which I’m delighted to do. Fortunately, my role is going to be made much easier because we’ve just appointed two deputies to work alongside me.

Shaun Robinson, Consultant Clinical Scientist at Imperial will be well known to many of you through his numerous publications in *ERP* and for his roles as an active Council member of the BSE and Co-chair of the Education Committee. Prof Paul Leeson from Oxford really requires no introduction. He is a very active researcher with many publications to his name and a very strong academic pedigree. I’m very excited to work alongside Shaun and Paul and we have many plans to take the Journal forward and increase its profile. So, look forward to an increased presence on social media, a revamped electronic table of contents with editorial comments about published manuscripts and video interviews with lead authors of important papers, to name but a few of our plans.

In June we published a synopsis of the 2021 Annual Advances in Contrast Ultrasound Conference [2]. This is a major event for anyone interested in Contrast Echo with both cutting edge and the latest contrast research presented. Now you can catch up with what’s new in this field from the comfort of your armchair. Enjoy and learn!

The June publications also contained a fascinating paper on the follow-up of patients with prosthetic heart valves [3]. It’s important food for thought because it highlights the huge workload this group of patients represents to most Echo departments and the low incidence of patients with complications. With the large backlog of patients requiring echocardiograms (especially post pandemic) this maybe an area for rationalisation—a personal view.

June also saw the final transfer of all previous ERP content to its new home on the Springer website. This provided me with a welcome opportunity to revisit earlier work including the BSE Guideline Document on the assessment of mitral valve disease [4]. This is such a comprehensive document that covers all aspects of mitral valve disease including many practical hints and tips. It is of course endorsed by the BSE, has been written by international experts in the field, and I think should be mandatory reading for all of us performing Echo on patients with mitral disease.

This month, we are publishing an updated Guideline from the BSE and British Heart Valve Society on clinical indications and triaging for adult TTE. As I’ve previously mentioned, all Echo departments are under huge pressure with large backlogs and staff shortages. This important paper provides key advice on how to provide incremental value to patients and standardise patient care. Essential reading for those who run Echo Departments, perform TTEs and refer patients for Echo. Need I say more?

In July, we are also publishing an interesting paper on an under diagnosed condition—mitral annular disjunction. The authors have reviewed the impact that this condition has on surgical outcomes following mitral valve surgery. The results are relevant to all Cardiothoracic Surgical Centres but to find out what the authors conclude—you will have to read the paper in *ERP*!

In future months, we have a range of fascinating research publications and important Guideline/Recommendation documents. So, keep your eye open for them by signing up for our monthly e-alerts (Table of contents): https://www.biomedcentral.com/add-article-alert?journalId=44156 [5]. And don’t forget that *ERP* is great Journal to submit your own papers to. Now that our initial technical issues have been resolved and we have a revamped editorial team, we are aiming for a rapid review and decision-making process. So, you will know quickly if your paper is suitable for publishing in this flagship Journal of the BSE.

We look forward to receiving and reading your submissions in the future.

Professor Mark Monaghan FBSE, (FRCP Hon), FESC, FACC.

Editor-in-Chief—*Echo Research and Practice.*

## Data Availability

Not applicable.
